# Changes in Water and Sewage Management after Communism: example of the Oder River Basin (Central Europe)

**DOI:** 10.1038/s41598-020-62957-1

**Published:** 2020-04-15

**Authors:** Włodzimierz Marszelewski, Adam Piasecki

**Affiliations:** 1 Department of Hydrology and Water Management, Faculty of Earth Sciences, Nicolaus Copernicus University in Toruń, Toruń, Poland; 2 Department of Geomatics and Cartography, Faculty of Earth Sciences, Nicolaus Copernicus University in Toruń, Toruń, Poland

**Keywords:** Environmental economics, Hydrology

## Abstract

This paper presents changes in water and sewage management in the cross-border Oder River basin in the period since the post-communist political and economic system transformation, including the period after Poland’s accession to the European Union. The Oder River basin, with an area of 124,000 km^2^, is the second largest basin in the Baltic Sea Basin, and therefore requires particular protection. It was emphasised that in the years 1989–2017, water withdrawal for production purposes considerably decreased (by 42%), as well as water withdrawal for exploitation of the water supply system (by 33%). The amount of sewage discharged to rivers was also reduced (by approximately 50%), and treatment technologies considerably improved. Changes in water and sewage management were presented in spatial form, i.e. by hydrographic regions of the Oder River basin. Particular attention was paid to changes in sewage management in cities. They involved among others the liquidation of mechanical treatment plants and a considerable increase in the number of cities with treatment plants with increased nutrient removal. The analysis of the effect of the changes in water and sewage management on the quality of the Oder River and Baltic Sea was also performed, and the rate of decrease in loads of contaminants most harmful to water ecosystems was determined. The role of European Union funds and national funds in the implementation of investments in the scope of water management was emphasised. Finally, attention was drawn to the need to intensify works for protecting waters in agricultural areas, which currently constitute the primary threat to their quality. Several top-priority tasks were also specified that should be implemented in the near future for the purpose of obtaining a good ecological state of waters in the Oder River basin pursuant to the Water Framework Directive.

## Introduction

A free market economy began to be introduced in Poland in 1989, entirely changing the approach to water management issues. It was decentralised, with a small contribution of private ownership (approximately 12%), in contrast to, for example, the Czech Republic, Portugal or Great Britain, where it reached 70–100%^1^. Sorting out water management began much later in Poland than in other European countries. France is a good example, where river contracts (RC), i.e. requalification programmes for rivers and other water bodies, were introduced already in the early 1980s^2^. Poland had no chance to participate in the project of countries of Western Europe that from 1975 set binding water quality targets for drinking water and other uses and limits on emissions. It also did not implement the Urban Waste Water Treatment Directive^3^ or Nitrates Directive in the early 1990s. An important breakthrough in the approach to water management occurred in the beginning of the 21^st^ century with the introduction of the Water Framework Directive (WFD). Numerous projects dedicated to implementing the WFD are currently underway both in Poland and in other European countries, including, among others, management plans, technical solutions, socio-economic and legal instruments, and recommendations for institutional restructuring^3,4^.

The three primary objectives of this paper are as follows:

1. analysis and assessment of changes in the scope of water withdrawal and sewage treatment in the Oder River basin (with particular consideration of cities) in the period of almost 30 years from the onset of the system transformation
2. evidencing the effect of the aforementioned changes on the quality of rivers and the Baltic Sea
3. formulating recommendations concerning future water and sewage management in the Oder River basin

The Oder is one of the largest rivers in the basin of the Baltic Sea, and an important trans-border river in Europe. The springs of the river are located in the Oder Highlands (Oderské vrchy) in the Czech Republic at a height of 634 m a.s.l. Its length is 854 km, including 742 km in the territory of Poland. Due to the variability of the longitudinal inclination, three characteristic sections of the river are designated, namely: upper (length 202 km, inclination from 7.2 to 0.33‰), middle (length 522 km, inclination from 0.28 to 0.19‰), and lower (length 130 km, inclination from 0.05 to 0.001‰). The border between Poland and Germany runs along the lower section of the Oder River. The mouth of the river to the Baltic Sea (through the Szczecin Lagoon) is located in the territory of Poland. The mean multiannual discharge of the Oder River is 567 m^3^·s^−1^. Its longest tributary is the Warta River (808 km), with a basin area of 54,500 km^2^, constituting as much as 44% of the entire area of the Oder River basin. Mean multiannual water discharge in the Warta River is 216 m^3^·s^−1^.

The Oder River basin is, with an area of 124,000 km^2^, the second largest in the Baltic Sea basin. It is 86% located in Poland, 8% in Germany, and 6% in the Czech Republic. The basin is located in the temperate climatic zone with strong influences of air masses from over the Atlantic Ocean. Mean air temperature in the years 1981–2010 was 9.0 °C, and shows an increasing tendency of 0.31 °C·10^−1^ years^5^.

## Methods

The analysis of changes in water management in the scope of water withdrawal and sewage treatment was based on data of the Central Statistical Office (CSO) in Warsaw. CSO collects data from all entities operating in the national economy whose activities include the collection, treatment, and delivery of water to local customers, collection and treatment of wastewater, emptying of outflow-free reservoirs and other bodies of water, and transport of liquid waste. The data are collected via the thorough observation method. The data were published in *Environment*^6^ yearbooks, and related to particular parts of the Oder River basin – hydrographic regions. This allowed for a detailed analysis of changes in the selected partial basins of Oder, and basins of its largest tributaries. Particular attention was paid to urban areas, due to their high importance for water quality. The above information provided the basis for the development of a database concerning the following elements:

- water withdrawal for the needs of the national economy and population by withdrawal sources (water collection to meet the needs of the national economy and population by collection sources or the quantity of water used to meet customer demand originating from individual sources or the water main network. In a statistical sense, this is the total of the water used for industrial purposes, farming and forestry as well as household use.)
- length of water supply and sewage networks (length of water supply and sewer networks (length of water supply in reference to cities includes systems where the local street water distribution network is at least 250 m long and at the same time serves 5 residential buildings, with at least 25 flats or 2 street outlets; length of sewer network includes systems where the street combined sewer network is at least 250 m long and from which there are at least 5 connections to residential buildings or yard inlets as well as cities with a precipitation water network, if the network is used for the discharge of household wastewater as well).
- percentage of people using the water supply network and sewage treatment plants (Data on population connected to water supply and sewage systems comprise population inhabiting residential buildings connected to a particular network as well as population using water supply systems via street and yard outlets and sewage system via sewage inlets)
- amount of water used by households (amount of water used by households corresponds to water usage levels via water mains supplying households and groups of households, both charged and uncharged usage, independent of any fees charged for this water in cities and rural areas).
- amount of treated sewage (with consideration of treatment type) and untreated sewage (sewage treated using a process of adjustment to environmental standards or other quality norms; untreated sewage – wastewater not purified via any process and released into surface bodies of water in original form).
- load of contaminants in treated sewage (e.g. pollutants loads in wastewater is the amount of pollutant in wastewater discharged in a given time unit and equals to the product of wastewater flow rate and pollutant concentration)
- number of sewage treatment plants with consideration of their type (four treatment plant types are considered: biological, chemical, mechanical, wastewater treatment plant with increased biogenic substance removal)
- financial expenditures for fixed assets for the purposes of water and sewage management (data on outlays on fixed assets for environmental protection and their tangible effects are presented in accordance with the Polish Statistical Classification concerning Activity and Equipment related to Environmental Protection introduced by the virtue of the regulation of the Council of Ministers of 2 March 1999 (O. J. No. 25, item 218). This classification was compiled on the basis of the ECE/UN Single European Standard Statistical Classification of Environmental Protection Activities and Facilities as well as the European System for the Collection of Economic Information on the Environment (SERIEE), implemented by the European Union (EUROSTAT). These data are consistent with data presented from 1996).
- consumption of mineral fertilisers (use of mineral fertilisers including mineral, calcium-based and calcium-magnesium fertilisers).

The source of the data was statistical surveys conducted by Statistics Poland based on the annual reports of, among other ministries, the General Directorate for Environmental Protection, the Institute of Environmental Protection – National Research Institute, and the Chief Office of Geodesy and Cartography. Additionally, data was taken from the following authorities: the Inspectorate of Environmental Protection, the State Sanitary Inspection and specialist services in the fields of hydrological and meteorological, geological, geodesic, forestry and nature protection. They permitted a detailed explanation of the analysed transformations, including from the perspective of water policy.

The positive effects of improving the water quality of the Oder River were documented based on an analysis of the variability of several ecologically key parameters: total nitrogen, total phosphorus and BOD^7^. In addition, these parameters were selected because long-term data was available for them, i.e. from the early 1990s to the present. In this period, monitoring studies methods have changed several times and it is difficult to conduct similar analyses for other parameters.

The collected data were subject to statistical analysis and geovisualisation. The statistical analysis determined average, minimum and maximum values. The linear regression method was used to examine the data (time series) in terms of statistically significant trends (α = 0.05).

## Results

### Water withdrawal

**Figure 1:**
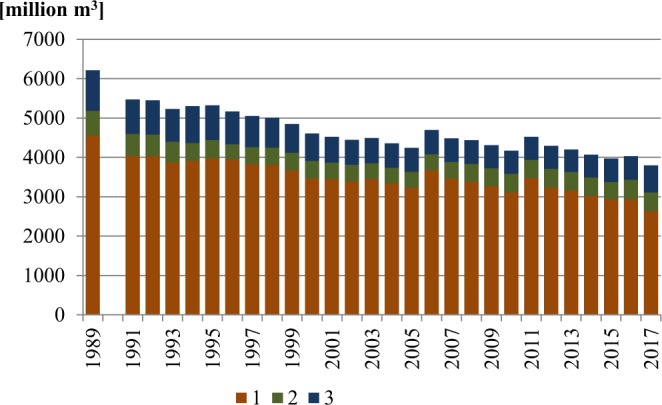
Water withdrawal for the needs of the national economy and population in the Oder River basin for the purposes of: 1 – production, 2 – agriculture and forestry, 3 – exploitation of the water supply system.

In the year the system transformation began (1989), total water withdrawal in the Oder River basin was 6.21 km^3^, including 4.55 km^3^ for production purposes, 1.03 km^3^ for the exploitation of water supply systems, and 0.63 km^3^ for the purposes of agriculture and forestry. In successive years, a statistically significant downward trend in water consumption was found. The directional coefficient of the built-in linear regression model indicates that each year there was an average decrease in water consumption of 57.484 million m^3^. In 2017, water consumption was only 61% of the 1989 volume (Fig. 1).

In the years 1989–2017, water withdrawal was reduced particularly for production purposes – from 4.55 to 2.63 km^3^, i.e. by 42%, as well as for the exploitation of the water supply system (from 1.03 to 0.69 km^3^, i.e. by 33%). Changes in water withdrawal for agriculture and forestry, in spite of its overall reduction from 0.63 to 0.48 km^3^ (i.e. by 24%), were not uniform. In the years 1989–2006, water withdrawal decreased (to 0.40 km^3^), and in the following years it slowly increased to 0.48 km^3^.

### Industrial and municipal sewage treatment

**Figure 2:**
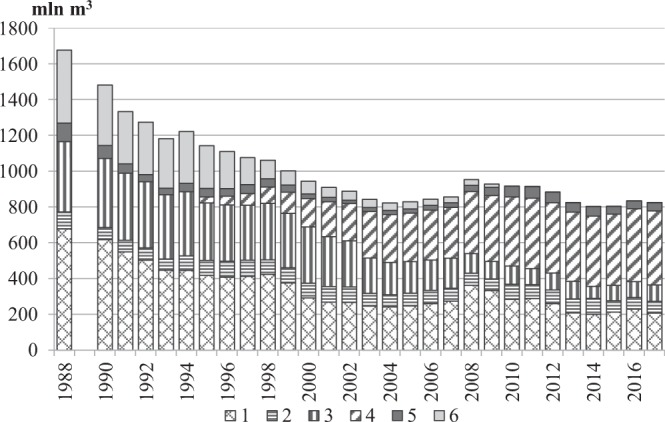
Changes in the amount of sewage discharged to rivers in the Oder River basin, and types of its treatment. Explanation: treated sewage (1 – mechanically, 2 – chemically, 3 – biologically, 4 – with increased nutrient removal); untreated sewage (5 – from production plants, 6 – from sewage networks).

Approximately a dozen main possibilities of water use have been recognised so far^8^. A strong dependency was also determined between the amount of water withdrawn and the amount of sewage. The highest amount of sewage originates from the production and municipal sector. The amount of agricultural sewage is difficult to determine because a considerable portion constitutes so-called non-point source pollution. Not all uses of water, however, directly contribute to generating sewage (e.g. sprinkling irrigation and watering, preservation of aquatic life). Moreover, a portion of water is subject to evaporation. Due to this, the amount of sewage is considerably smaller than the amount of withdrawn water. In the case of the Oder River basin, the volume of sewage introduced to rivers in the years 1989–1991 constituted 26.9–24.3% of the volume of water withdrawal, and in the years 2015–2017 approximately 21.5%. Like water consumption, the amount of wastewater in the analysed period showed a downward trend. However, this was less than half the size, at an average of 20.7 million m^3^ per year (Fig. 2). For the environment, however, the degree of treatment of sewage is a more important parameter than its amount. It depends on the technologies applied in sewage treatment plants. The technologies changed depending on the level of development of science and technology, as well as on the available financial resources.

In the Oder River basin, three methods of sewage treatment had been applied up until 1994, namely: physical, chemical, and biological. In 1995, the introduction of the modern method with increased biological nutrient removal commenced. Throughout the period 1989–2017, several characteristic tendencies of changes in sewage treatment were observed (Fig. 2):

- a strong decrease in the amount of sewage subject to physical treatment (from 0.68 to 0.21 km^3^).
- a strong decrease in the amount of sewage subject to biological treatment (from 0.39 to 0.09 km^3^).
- a slight decrease in the amount of sewage subject to chemical treatment (from 0.10 to 0.07 km^3^).
- a strong increase in the amount of treatment with increased nutrient removal from 1995 (from 0.03 to 0.41 km^3^).

In the years 1988–2017, the amount of untreated sewage was considerably reduced, from 510 to 50 million m^3^, with sewage from sewage networks constituting the majority of al sewage. Currently, almost 100% is treated, and only 0.04 km^3^ of sewage from production plants remains untreated (Fig. 2).

Changes in the amount and types of sewage treatment occurred in different ways in particular parts of the Oder River basin, where eight main hydrographic regions were designated. The names and numbers of the regions (from 1 to 8), as well as the most important data regarding them are presented in Table 1.

**Table 1:** Hydrographic regions of the Oder River basin. Explanation: AL – Arable land, PM – Pastures and meadows, F – Forests, UA – Urban area.

No.	Name of hydrographic region	S [km^2^]	Land use [%]				People			Number of city
			AL	PM	F	UA	total [in millions]	pop. density [/km^2^]	in city [%]	
I	Oder from springs to Nysa Kłodzka and Nysa Kłodzka basin	17,993	38.7	7.8	31.0	7.7	3.80	209.3	72.8	89
II	Oder from the mouth of Nysa Kłodzka to the mouth of Bober	22,932	51.3	6.5	29.5	5.7	3.9	170.1	63.5	114
III	Bober basin	5,870	29.7	6.5	45.7	6.1	1	168.2	64.1	44
IV	Odera from the mouth of Bober to the mouth of Warta	6,831	24.5	9.6	52.1	5.0	0.4	58.1	58.1	23
V	Warta from springs to the mouth of Prosna, and Prosna basin	20,721	49.1	10.4	22.7	5.6	3.7	180.0	57.1	85
VI	Warta from the mouth of Prosna To the mouth to Oder	16,469	50.1	7.0	30.7	4.4	2.7	166.8	59.1	89
VII	Noteć basin	17,289	39.2	8.4	40.4	2.4	1.8	106.8	58.0	79
VIII	Oder from the mouth of Warta to the mouth to the Szczecin Lagoon	10,880	47.1	10.0	29.7	3.9	1	89.1	72.6	31

**Figure 3:**
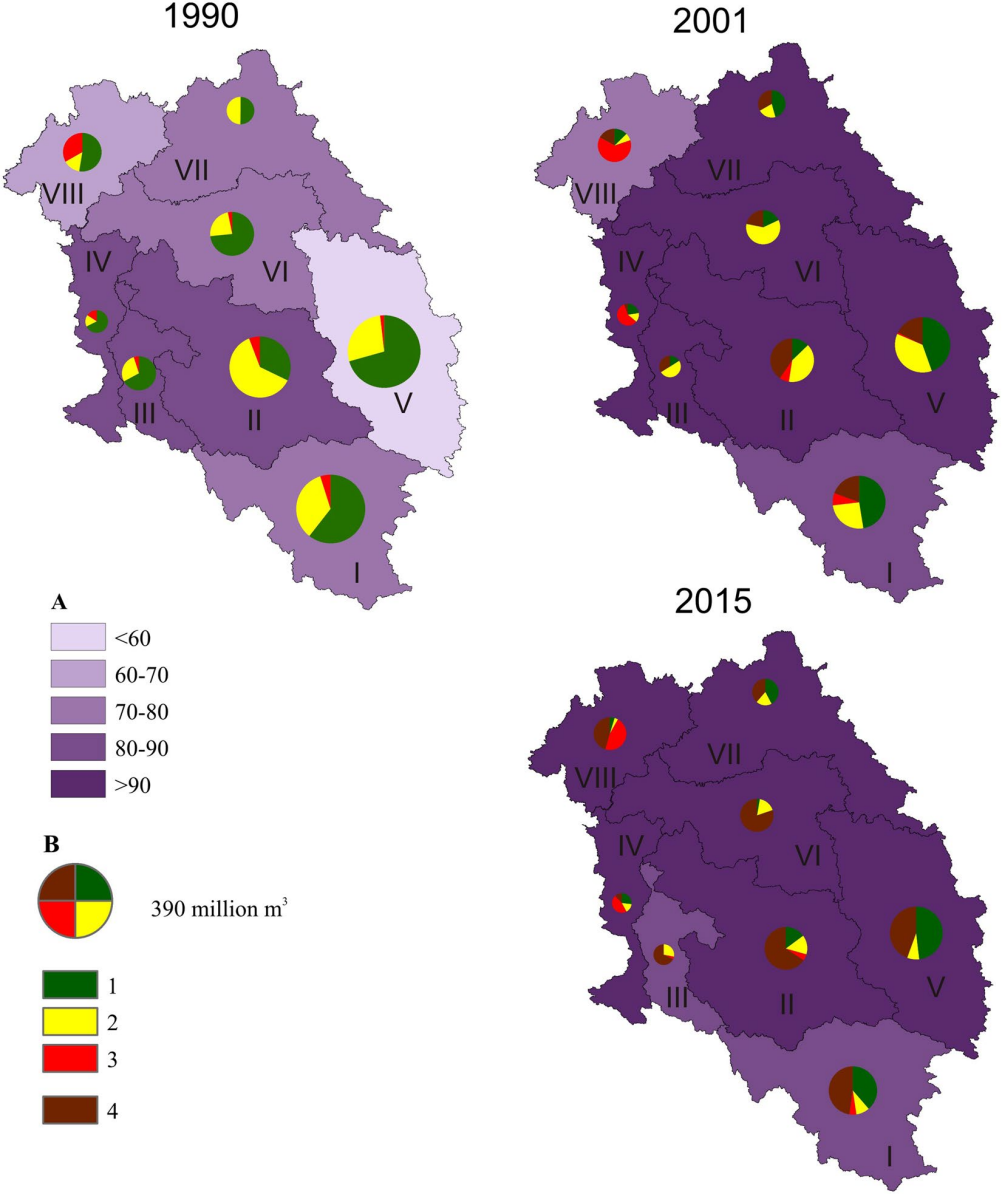
Changes in the amount of treated sewage and types of sewage treatment in hydrographic regions of the Oder River basin. (**A**) Percent of treated sewage; (**B**) amount of treated sewage and type of treatment: 1 – mechanical treatment, 2 – biological treatment, 3 – chemical treatment, 4 – treatment with increased nutrient removal.

In 1990, particular hydrographic regions of the Oder River basin had a high variability of contribution of treated sewage in the total amount of sewage: from <60% in region 5 to 80–90% in regions 2, 3, and 4 (Table 1, Fig. 3). Physical sewage treatment was predominant in all regions. Its contribution in region 6 was as much as 80.5%. Biological sewage treatment was predominant in region 2 (62%), and was applied to the lowest degree in regions 8 and 4 (14% and 17%).

In the years 1990–2001 in the analysed regions, the contribution of treated sewage in the total amount of sewage levelled off. In six regions, the share of treated sewage increased by >90%. It was lower only in regions 1 and 8, where it equalled, respectively, 80–90% and 70–80% (Fig. 3). In all regions, the amount of sewage subject to physical treatment considerably decreased (by half on average), as well as the amount of sewage subject to biological treatment. Mechanical and biological sewage treatment was replaced with treatment with increased nutrient removal. In some regions (e.g. 3 and 2), this modern treatment method covered more than 40% of sewage already in 2001.

In 2015, the share of treated sewage in the total amount of sewage averaged 94.6%, and only in two regions (1 and 3) was it somewhat lower than 90%. Sewage treatment with increased nutrient removal was dominant almost everywhere. In regions 2, 3 and 6, it was, respectively, 66, 68 and 83%.

### Sewage treatment in cities

The Oder River basin currently includes 386 cities within the territory of Poland (19 more than in 1990). The vast majority (more than 60%) are small cities with a population of <10,000. The total number of residents of all the cities, however, is high, at 8.4 million. Due to this, sewage treatment in the cities of the Oder River basin is a very important element of sewage management.

**Figure 4:**
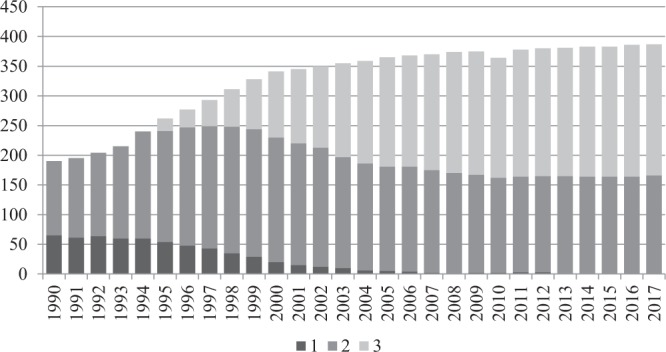
Changes in the number of cities with sewage treatment plants and types of sewage treatment technology in the years 1990–2017. Explanation: 1 – mechanical treatment plants; 2 – biological treatment plants; 3 – treatment plants with increased nutrient removal.

In the years 1990–2017, the number of cities with sewage treatment plants increased considerably. The technology, and therefore efficiency of sewage treatment, also substantially changed. In 1990, waste treatment plants supported only 190 out of the 367 cities existing at the time. In 2017, almost all cities (99.2%) had treatment plants (Fig. 4). The fastest increase in launching new sewage treatment plants (on average 13 cities per year) occurred in the years 1992–2002. It is worth emphasising that irrespective of the increase in the number of cities with sewage treatment plants, in the majority of the remaining cities the treatment plants were modernised. This, however, is a separate issue not discussed in this paper. These positive changes occurred despite a decrease in total number of treatment plants. Mechanical treatment plants and outdated biological treatment plants were closed. They were replaced by modern treatment plants with enhanced nutrient removal and much higher throughputs.

In the years 1990–2017, three main tendencies concerning cities were observed, depending on the sewage treatment technology (Fig. 4):

- a decrease in the number of cities with physical sewage treatment plants from 65 (1990) to 1 (2013) as a result of launching new treatment plants in cities and introducing new technologies.
- an increase in the number of cities with biological treatment plants in the period 1990–1997, followed by a decrease (in the years 1998–2017).
- an increase in the number of cities with treatment plants with increased nutrient removal in the period 1995–2017.

**Figure 5:**
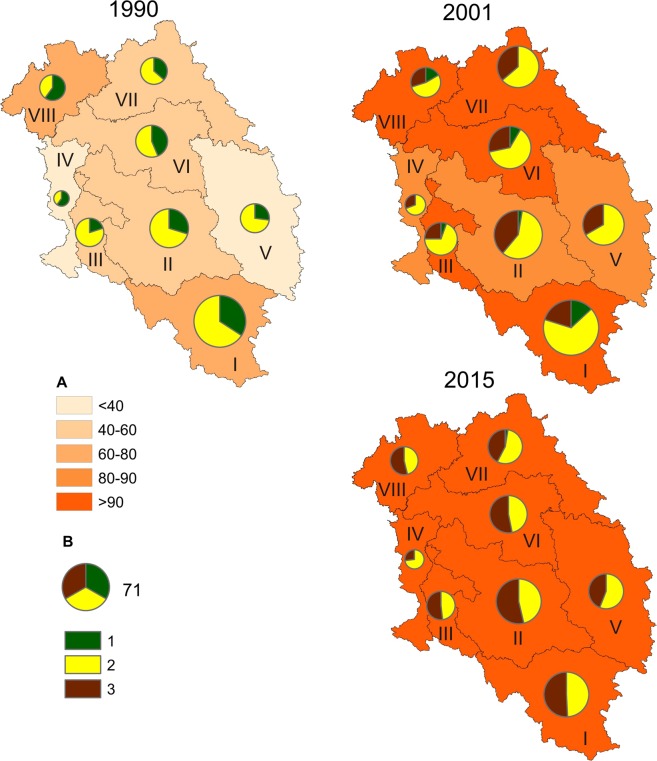
Changes in the number of cities with sewage treatment plants and types of treatment plants in hydrographic regions of the Oder River basin. (**A**) Percent of cities supported by sewage treatment plants; (**B**) number and type of sewage treatment plants: 1 – physical treatment plants, 2 – biological treatment plants, 3 – treatment plants with increased nutrient removal.

The aforementioned changes had a different course in particular hydrographic regions of the Oder River basin. In 1990, the share of cities supported by sewage treatment plants was variable, from <40% in regions 4 and 5 to 60–80% in regions 1 and 8 (Fig. 5). In almost all regions (except for 4 and 8), the largest single group of cities was those with biological sewage treatment plants.

In 2001, the number of cities supported by sewage treatment plants increased considerably throughout the analysed area. In regions 1,3,6,7 and 8, the share of such cities constituted more than 90% of their total number. In cities of all regions, biological sewage treatment was predominant, and 37% of cities were supported by treatment plants with increased nutrient removal. The role of physical treatment substantially decreased. It did not occur at all in several regions (4, 5, 7).

In 2015, almost all cities were equipped with treatment plants (Fig. 5). The highest number of cities (219) had treatment plants with increased nutrient removal or biological treatment plants (163). A physical treatment plant functioned in only one city.

### Effect of water and sewage management changes on Oder River and Baltic Sea water quality

A decrease in the amount of sewage and increase in the reduction of contaminants in sewage improved the quality of rivers. The concentration of all chemical and biological substances decreased, and physical parameters monitored in National Environmental Monitoring studies improved. The changes were (and still are) very evident not only in the lower section of the Oder River before its inflow to the Szczecin Lagoon, but also in all its tributaries. The lower section of the Oder River is recognised as the most representative, however, especially since water quality along this section determines the load of contaminants introduced to the Baltic Sea from the entire basin.

**Figure 6:**
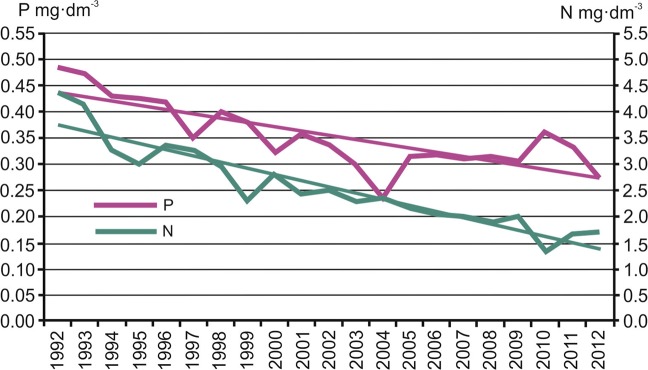
Changes in mean nitrogen and mean phosphorus concentrations in the Oder River in the municipality of Krajnik. Data according to the National Environmental Monitoring (2015).

Since the early 1990s, nutrient concentrations have decreased significantly. Regression analysis of nitrogen and phosphorus values show a statistically significant downward trend. In the municipality of Krajnik (59 km before the inflow of the Oder to the Szczecin Lagoon), the mean annual value of total nitrogen concentration decreased from 4 l.82 to 2 l.78 mg·dm^−3^ (Fig. 6).

Nitrogen concentration did not decrease evenly over the period. It was considerably faster in the first part of the period, and in the years 1992–2004 averaged 0.184 mg·dm^−3^. After 2005, nitrogen concentrations stabilised.

A similar tendency of changes was also observed for phosphorus. Its concentration decreased from 0.434 to 0.169 mg·dm^−3^ in the years 1992–2012 (Fig. 6). As with nitrogen, the fastest decrease in phosphorus concentration occurred in the first years of the water and sewage management being improved. In the period 1992–1999, phosphorus concentration decreased every year by an average of 0.026 mg·dm^−3^, and in the next period by only 0.008 mg·dm^−3^.

The decrease in total phosphorus and total nitrogen concentrations was not systematic in each consecutive year. It was largely dependent on the discharge volume of the Oder River in a given year.

As mentioned above, the pollution of rivers was reduced throughout the basin. An example is a decrease in the concentration of biochemical oxygen demand (BOD) – an indicator of water pollution with municipal sewage – in the Oder River in Wrocław. In the 1980s, BOD concentration averaged 9.2 mg O_2_·dm^−3^ annually. In 1992–2016 there was a statistically significant downward trend in BOD concentration; during this period, the total BOD decrease was over 6 mg O_2_·dm^−3^ (from 9.10 to 2.00 mg O_2_·dm^−3^). As in previous cases, the fastest decrease in BOD concentration occurred at the beginning of the analysed period, i.e. in the years 1992–2001, when it averaged 0.72 mg O_2_·dm^−3^ per year. Notice also that from 2005, BOD concentration in the Oder River (Wrocław) was lower than the threshold value determined for class I and abiotic type 21 signifying a great lowland river (according to the EU Water Framework Directive).

**Figure 7:**
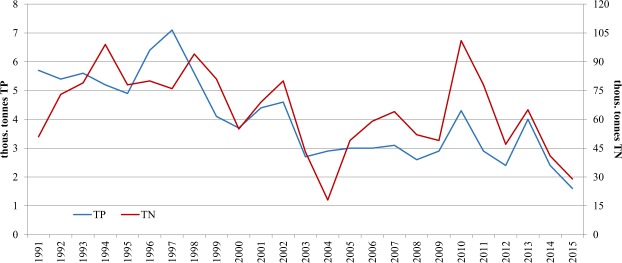
Total nitrogen and total phosphorus load introduced to the Baltic Sea through the Oder River in the years 1990–2015. Elaboration based on data of the Central Statistical Office and IMGW-PIB.

Pollutant loads depend both on their concentration and on water volume. Due to this, a decrease in concentrations has not always been accompanied by a reduction in pollution load. In the case of total nitrogen load, years of considerable reduction alternated with years of high loads (Fig. 7).

In spite of an evident tendency for a decrease in total nitrogen load introduced in the Baltic Sea in the years 1990–2015, there were years in which the load was highest and did not correspond with the general direction of changes. Such a situation occurred in 2010, when water outflow in the Oder River was the highest in 30 years, at 25.54 million m^3^, i.e. more than 8 million m^3^ more than the mean annual value (according to data of IMGW-PIB). It is therefore not surprising that the total nitrogen load introduced to the Baltic Sea in 2010 was more than 100,000 tonnes, and was among the highest in history. The lowest TN load (26,880 tonnes) was introduced to the sea in 2015.

The rate of decrease in total phosphorus load introduced to the sea was faster and more uniform (Fig. 7). The highest TP load was introduced in 1997 (7,110 tonnes), although water outflow was approximately 12% lower than in 2010. In this case, phosphorus concentration was of crucial importance. In 1997 it was more than double what it was in 2010 (Fig. 6). The lowest amount of total phosphorus (1,520 tonnes) was introduced to the Baltic Sea in 2015.

Similar changes occurred in the biochemical oxygen demand load (BOD). The rate of decrease in the BOD load, however, was lower than the course of changes in TN and TP loads. The highest BOD load (118,230 tonnes) occurred in 1997, and the lowest (28,950 tonnes) in 2015. The mean rate of decrease in the BOD load introduced to the Baltic Sea was 1,730 tonnes year^−1^.

## Discussion

The Oder River basin was the area posing the greatest threat to the Baltic Sea in the second half of the 19th century and the first half of the 20th century. This was associated with the rapidly growing industry, increasing urbanisation and agricultural development of that time. As a result, in the years 1880–1940 the nutrient loads introduced into the Baltic Sea by the Oder increased from 34,000 to 50,000 tonnes TN year^−1^ and from 2,600 to 3,900 tonnes TP year^−1^. During that period in Europe, higher nutrient loads were only introduced by the rivers in the North Sea catchment area: the Rhine and the Elbe^9^. In 1880, about 90% of TN and TP in the Oder waters came from spatially disparate sources of pollution – septic tanks in particular. In the following years, nitrogen and phosphorus water pollution from municipal sewage systems increased steadily, and by 1940 already constituted over 40% of total nutrient load. In the entire Oder river basin, as in neighbouring basins, there began to dominate nitrogen and phosphorus pollution from urban centres, which had a total population of 19.7 million in 1940. Even then, the Oder basin was the most populous of all Baltic river basins. An adverse phenomenon was the decrease in nutrient removal in the treatment plants of that time. In the years 1880–1940, removal decreased from approximately 80 to 60% for nitrogen, and from approximately 86 to 66% for phosphorus^9^. These facts largely explain the highly negative impact of the Oder river basin on the Baltic Sea after the Second World War. The economic functioning of cities and industry in the Odra River basin under communism did not favour effective water protection measures. Hence, the decision to thoroughly redevelop municipal infrastructure as early as the 1990s (initially with the support of pre-accession funds) should be considered both extraordinarily appropriate and extremely costly. The cost of reducing the amount of nitrogen and phosphorus introduced into the Baltic Sea from Poland, including the Oder river basin, was universally calculated to be the highest of all countries in the Baltic catchment^10^. Currently, as already mentioned, the most important problem is how to limit the supply of nutrients from diffuse sources. The method developed as part of the EUROHARP project^11^ may be helpful in this.

**Figure 8:**
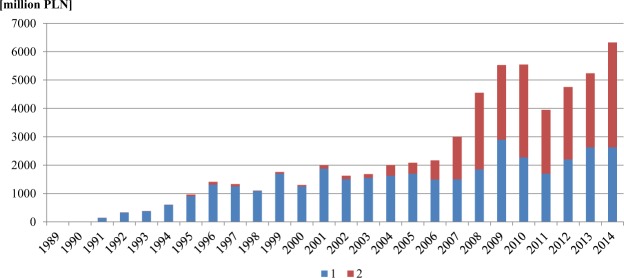
Financing tasks in the scope of the environment and water management (1 PLN = 0.24 EUR). 1 – Domestic resources, 2 – European resources. Source: data of the National Fund for Environmental Protection and Water Management.

In 1989, the National Fund for Environmental Protection and Water Management (NFEPWM) was founded, as well as the Regional Fund for Environmental Protection and Water Management (RFPWM). One of the primary objectives of the institutions was and still is granting financial support to undertakings related to water and sewage management, one of the most neglected sectors at the end of the 20^th^ century. The NFEPWM and RFEPWM were also tasked with the efficient use of financial resources of the European Union, Norwegian Financial Mechanism, and other sources, including domestic ones. In the years 1993–2014, NFEPWM and regional RFEPWM financed more than 20,000 agreements on subsidies for investments in the scope of the environment and water management. The total value of all investments was 135 billion PLN, i.e. approximately 31.4 billion EUR (including approximately 40% of the amount that was invested in the Oder basin area). In the same period, co-financing of investments by NFEPWM equalled 60 billion PLN, including 23.4 billion PLN from European resources. The share of European resources rapidly increased after Poland’s accession to the European Union (Fig. 8). Out of total resources, the greatest amount (more than 40%) was granted for financing projects related to the protection of waters and water management.

An exceptionally high increase in financing of projects related to the protection of waters and water management after 1989 in the Oder River basin contributed to, among others:

- modernisation and construction of new sewage treatment plants, including household sewage treatment plants,
- liquidation of small and technologically obsolete treatment plants (particularly mechanical ones),
- modernisation of the sewage system in cities, and fast expansion of the sewage system in rural areas.

Mean water consumption in the Oder River basin over the recent five years of 2013–2017 was 4.01 km^3^, which constitutes approximately 33% of total renewable freshwater resources available. The percentage, called water exploitation index plus (WEI + ), suggests that the water resources of the Oder River basin remain “under stress”, because the value of WEI+ exceeds 20%^12^. The Oder River basin is among the most threatened basins in Europe in those terms. In the period April–June 2015, only 10 other basins in Europe were characterised by a WEI + higher than that for the Oder River (among others, the Douro, Ebro, Guadiana and Tagus on the Iberian Peninsula, the Pinios in Greece, the Ems/Weser, and basins in Sicily), and in the period October–December 2015 only two: the Ems/Weser and river basins of the Attica River Basin District^13^.

Since 1989, the transformation processes and socio-political processes in Poland, as well as the development of modern technologies, have led to a decrease in water consumption in households and the production sector. Investments in modern water-saving technologies proved to be the most important in terms of limiting water exploitation and pollution. This is confirmed by conclusions drawn by Flörke *et al*.^14^ regarding water consumption at a global scale since 1950. In the case of the Oder River basin, another important factor contributing to saving water was a more than threefold increase in the price of cold water and sewage. The price of 1 m^3^ of cold water per individual recipient increased from 1.28 PLN to 3.92 PLN in the years 1999–2017 (while total inflation at the time was 32%)^15^. On the other hand, an increase in water consumption is observed during hot summers in some years (e.g. in 2015). This fact is closely related to increasingly higher air temperature in the period of climatic transformations, which was also recorded in southern Europe^4^.

On the background of the overall decrease in water withdrawal, a somewhat different tendency occurs in the case of water withdrawal for agriculture and forestry. A slow increase in water consumption is observed in agriculture, probably related to climatic changes. An increase in air temperature of 0.33 °C·10 years^−1^ in the years 1961–2010^5^ and sunshine duration in the years 1999–2013 from 1571 to 1697 hours on average^16^ in this part of Europe contributes to an increasing need to irrigate agricultural areas, including sprinkling irrigation.

In recent years the number of groundwater exploitation sites for irrigation systems has increased quite substantially across parts of the Oder river basin (regions V, VI, VII – Table 1). This is a very dangerous trend due to poor control over the amount of water collected in this manner. This is a novel problem that also happens to be complex in nature and not extensively studied in Poland. Hence, the present study raises this issue in order to signal the existence of a problem. The authors of the present study are already conducting research in this area. On the other hand, it is necessary to note major limits on the quantity of surface water collected for agricultural purposes. As late as the 1980s the number of water collection sites on lakes and rivers was large and the water was used to irrigate crops, which did contribute directly to a lowering of water levels in lakes. Today the direct collection of surface water is rare due to significant declines in water resources resulting from climate change. While the Oder’s average atmospheric precipitation total for its drainage basin remains similar to that in years past at 570 mm, the decline in surface water resources is readily observable. This is caused by climate change and more precisely increased evaporation and reduced retention in the winter months due to a lack of snow cover.

**Figure 9:**
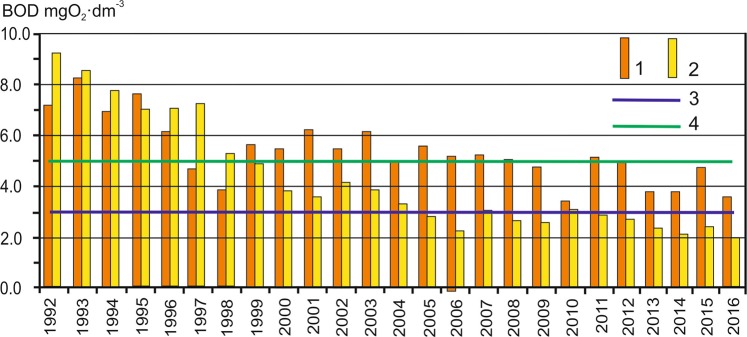
Mean annual BOD5 concentration in Chałupki (1) and Wrocław (2). The violet line signifies the threshold for class I, and green for class II.

In the case of international basins, another important element is the quality of water flowing into the territory of a given country. Unfortunately, concentrations of some water quality parameters point to the Oder River being already heavily polluted at the border of Czech Republic and Poland. One example is the concentration of BOD5 in the municipality of Chałupki. Since 1999, every year it is considerably higher than the concentration of BOD5 in Wrocław lower downstream (Fig. 9).

In the years 1991–2015, the number of cities supported by sewage treatment plants more than doubled. In the same period, the share of population of cities benefitting from treatment plants in the total number of residents of cities increased from 55.5% in 1991 to 95.6% in 2015. The level approximates to that in, among others, Austria, Germany and Switzerland, and is somewhat lower than in Holland or Great Britain^17^. In cities, sewage treatment plants with enhanced nutrient removal predominate. Most engage in recycling and energy recovery. Due to this, the largest cities of the Oder River basin (Łódź and Wrocław) were designated as “water-efficient cities”, although they already meet (along with other cities) the majority of conditions specified for “resource-efficient and adaptive cities”^18^.

In the case of international basins, an important element of water and sewage management is cooperation between neighbouring countries, on both the national and regional (local) level. An interesting example of Polish–German partner cooperation in the Oder River basin is a shared sewage treatment plant for two cities located on either side of Nysa Łużycka: Gubin (PL) and Guben (DE). The legal aspects of cooperation between EU countries, particularly in the aspect of the Water Framework Directive^19^, were discussed among others by Keessen^20^, and examples of integrated water management in French–Italian basins were presented by Scaduto^2^.

After 1989, total phosphorus emission throughout the Oder River basin decreased considerably. In the years 1995–2015, P emission into the Oder basin declined by approximately 2,500 tonnes year^−1^, and in a longer period (1985–2015) it decreased by more than 9,000 tonnes year^−1^ ^21^. Total amount of sewage also decreased by more than 50%, and the technology of its treatment substantially improved. Nonetheless, a rather slow decrease in contaminant concentrations in rivers and loads of contaminants introduced to the Baltic Sea is observed. This particularly concerns TP and TN. This is in spite of an almost complete reduction in contaminants from urban areas. This suggests that areal (agricultural areas) and dispersed – point pollution sources in rural areas – still remain an unresolved problem. Excess N and P from the aforementioned sources is supplied to surface waters. The primary factors contributing to an increase in N and P losses from agricultural land in the Oder River basin include, among others^21^: excessive use of mineral and natural fertilisers; greater congestion of concentrated pig farms, along with excessive use or improper storage of manure; an increase in meliorated areas; a decrease in the surface area of grasslands, and unfavourable changes in the consolidation (congestion) of the bedrock. It should be emphasised that a systematic increase in the use of mineral fertilisers has been observed since the beginning of the 1990s^22^. Problems with limiting nutrient inflow from diffuse sources were also documented in the case of the North Sea, Northern Adriatic, and North-Western Black Sea Shelf. It was determined that there is an evident policy success for point sources, notably for P in the Baltic and North Seas, but the reduction of diffuse sources is more problematic^23^. The emission and loads of nitrogen and phosphorus in the Oder River basin generally decreased. They are lower than in other rivers in Central Europe, and the long-term trends of their changes are similar to those observed in the Danube River^24,25^. A further decrease in mineral contaminants supplied to surface waters should occur in the upcoming years. This particularly concerns nitrogen. This is related to the new approach (since 2017) to the implementation of the Nitrate Directive in Poland (Council Directive 91/676/EEC of 12 December 1991). The entire territory of the country is currently considered as a Nitrate Vulnerable Zone. This entails the need to introduce a number of strict rules relating among others to fertilisation^26^.

Changes similar to those in the case of the Oder River were also documented in the Elbe River basin. The analysis of water quality changes in the Czech part of the Elbe River basin showed that the majority of the basin area experienced important improvements in water quality during the 1990s. The trends in surface water quality stem from the general socio-economic changes in the country after major political changes in 1989. The economic transition of the country, adoption of new legislative measures, as well as accession to the EU resulted in structural changes influencing water pollution. The rapid decline in sewage emissions from industrial and municipal sources resulted in the improvement of water quality in a major part of the Elbe River basin area^27^.

The issue of water management in reference to settlement in rural areas is comprehensive and complicated. The very evident, positive quantitative and qualitative evolution of the water and sewage infrastructure in those areas should be emphasised. The dynamics and total increase in the length of the sewage and water supply network were higher than in cities. The percentage of people using the sewage system, however, is still approximately 41.3%. The equivalent result for the water supply system exceeds 85.1%. As a result, many households use individual sewage management systems. The most commonly applied solution is closed-drainage reservoirs (periodically emptied). The solution is commonly criticised for, among others, the lack of provision of sewage neutralisation, and its periodical retention; improper construction (lack of tightness) and exploitation (rare emptying)^28^. In recent years, among others due to financing from EU resources, the aforementioned reservoirs have been replaced with household sewage treatment plants. Unfortunately, installations with a septic tank and filter drainage are the most frequently applied technology. According to many authors^29,30^, such installations constitute a serious threat to the quality of surface waters and groundwaters. The systems are currently becoming undesirable, and are even banned in some EU member states^30^.

A debatable issue, however, is the quality of statistical data concerning water management in rural areas. The way of monitoring water consumption and sewage management seems rather inaccurate. According to the official statistics, the level of sewage treatment in rural areas is very high (more than 99%). The Report of the Supreme Audit Office (SAO)^31^ of 2017 concerning rural communes of the Lubuskie Voivodeship (located in the Oder River basin) presents a number of irregularities in the scope. The correctness and effectiveness of the supervision over the frequency of emptying closed-drainage reservoirs from liquid sewage was evaluated very negatively. The supervision over the functioning of household sewage treatment plants was evaluated equally critically. The amount of treated liquid sewage in the inspected communes on average corresponded to 55.5%, and in an extreme case only 4.5% of the amount of water used for municipal purposes. The method of removal of the remaining amount of sewage (corresponding to a total volume of 4.1 million m³ of consumed water) generated in the area of the communes is unknown. Moreover, it was evidenced that 7 out of 20 inspected communes had failed to observe their obligation to conduct a register of crossed-drainage reservoirs, and the same was true of the obligation to control the functioning of household sewage treatment plants (in 17 out of 20 communes).

According to data of HELCOM^32^, mean TN concentration in the Oder River in 2014 was the highest among the seven largest basins of the Baltic Sea, and equalled 3.087 mg∙dm^−3^, and mean TP concentration of 0.177 mg∙dm^−3^ was the second highest. However, considering the fact that the majority of the TN and TP load to the Baltic is of anthropogenic origin, the size of population in the basin contributing to the pollution of the sea should be considered. The amounts of TN and TP per resident introduced to the Baltic from the Oder River basin in 2014 averaged, respectively, 2.76 kg and 0.16 kg. The values are the lower than in any of the other basins. Nutrient concentrations in the Oder River and all the remaining rivers in the Baltic Sea basin need to be further radically reduced. It will be possible in the case of the further development of the needs and objectives stipulated in the WFD^33^.

The Water Framework Directive has become an inspiration for the rapid improvement of wastewater management, especially in those countries that joined the EU in 2004^34–37^. Despite major financial and investment efforts, there is a concern that the goal of achieving good water status in EU river basins by 2027 (including the Oder river basin) will not be achieved. To achieve this goal it is recommended, among others^38^: to introduce innovative monitoring and assessment methods, to improve diagnosis of the causes of water quality degradation, to focus financial resources on reducing the main causes of water quality degradation, to demonstrate the various benefits of extending sewage management to all water users, and to increase the cohesion of the policy and its integration with other sectors, especially agriculture.

Considering the high costs related to water protection and management, the question arises of whether and what potential ecological benefits are gained by societies through the implementation of the WFD. Research on the subject was conducted among others in the scope of an interdisciplinary project in the Werra River basin in Germany^39^. The implementation of the WFD has brought about a number of socioeconomic benefits to the Oder river drainage basin. Access to water and sewer services has increased, thus increasing the quality of life for area residents. Care for the natural environment has also increased. Modernized technologies of water treatment including reduced use of chlorine in favor of chlorine dioxide and ozonation as well as filters with active carbon. The expansion of water and wastewater treatment infrastructure has also increased the investment attractiveness of rural and suburban areas. This is one reason why suburban and rural areas are experiencing more development and land use changes from agriculture to housing. The WFD has also introduced a number of limitations, especially on agriculture. Limits on fertiliser use have helped change the way arable land is used in some instances – especially parcels situated in close proximity to surface waters and parcels characterized by large relief gradients. Areas not considered to be fertile are increasingly being reforested.

## Conclusion

The response of water and sewage management to the political system transformation is one of the best examples of positive and extensive changes that occurred in the central part of Europe after 1989. The changes concern an exceptional element of the environment, namely water. The impetus for commencing radical changes in water and sewage management was pre-accession funds, and then EU subsidies or grants. Nonetheless, negligence in the scope proved to be so vast that one of the primary objectives of the Water Framework Directive was not implemented, namely good state of waters by 2015. For the implementation of the objective to be possible in the upcoming years, it is necessary to introduce changes in several areas of water and sewage management, and implement several important tasks, including among others:

- limiting agricultural pollutants (nutrients) supplied to waters, i.e. nitrogen and phosphorus; in the case of nitrogen, proper implementation of the Nitrate Directive is necessary; similar solutions should be developed and implemented for phosphates,
- drawing more attention to the problem of decreasing water resources in the eastern part of the Oder River basin confirmed by a decrease in mean annual values of unitary flow to less than 2.5 dm^3^·s^−1^·km^2^, and considerable acceleration of works aimed at an increase in water retention,
- intensification of the construction of sewage networks in rural areas, and introduction of the requirement to liquidate closed-drainage reservoirs and to simultaneously replace them with household sewage treatment plants,
- facilitation of the process of water and sewage management by self-governments, and intensification of their control in the scope of observing their statutory obligations resulting from Polish and European water law.
- undertaking actions, together with the Federal Republic of Germany and Czech Republic, aimed at obtaining additional resources from the EU for the improvement of the cleanliness of the Oder River; most of the resources should be spent on financing the aforementioned actions.

The data and situations presented in this article may provide not only an incentive to undertake similar projects aimed at organising sewage management in other areas, but also evidence of achievable success in several areas, namely: improving the quality of surface and groundwater in catchments and river basins, improving residents’ quality of life, renaturalising rivers and lakes alongside increasing biodiversity, and reducing negative impacts on the seas and oceans. Similar projects can be implemented, though they will require sufficient funding and public acceptance. In the case of international river basins, it also requires reconciliation and cooperation between countries and/or regions. The most important element is undoubtedly residents’ ecological awareness, which should be developed from an early age.

The improvement of water and sewage management in the Oder River basin will be particularly possible if appropriate financing is provided. In spite of dynamic development, Poland is not able to deal with the task on its own within a short time-frame. Therefore, currently, in the period of preliminary works on the new EU budget perspective for the years 2021–2027, it is important to lobby for appropriate funds, especially because various projects are being implemented in the protection and sustainable management of water resources in the European Union, as well as in the neighbouring countries. Properly developed water and sewage management is a key element of sustainable development, and constitutes a strategic objective of the EU. Therefore, the statement included in the title of a workshop a dozen years ago seems very accurate in this case: “Europe aims to bridge science and water management”.
